# The impact of TcdB conformational dynamics on vaccination against *Clostridioides difficile*

**DOI:** 10.1371/journal.ppat.1014627

**Published:** 2026-09-18

**Authors:** Megan L. Kempher, Tyler M. Shadid, Gillian A. Lang, Sydney T. Honold, Jordan N. May, Elizabeth J. Hall, Jason L. Larabee, Mark L. Lang, Jimmy D. Ballard

**Affiliations:** 1 Department of Microbiology and Immunology, University of Oklahoma Health Sciences Center, Oklahoma City, Oklahoma, United States of America; 2 Department of Molecular Microbiology and Immunology, University of Texas at San Antonio, San Antonio, Texas, United States of America; Texas A&M University, UNITED STATES OF AMERICA

## Abstract

*Clostridioides difficile* TcdB is a multidomain ~270 kDa protein that adopts at least two distinct structural conformations influencing receptor binding, sensitivity to bile salts, and antibody reactivity. Data from the current study suggest TcdB may benefit from conformational flexibility by avoiding neutralization by vaccine induced antibodies. Experiments further demonstrate that targeting a single TcdB conformation overcomes the toxin’s ability to evade antibody neutralization. In the first series of experiments, mice were administered TcdB engineered with a 19 amino acid deletion and two site specific mutations that, in combination, brought toxicity to undetectable levels. This TcdB formulation drove robust antibody and recall responses that protected mice from weight loss but not damage to the intestines. Though all the treated mice generated similar anti-TcdB titers, only about half of the mice produced detectable neutralizing antibodies to TcdB’s open conformation. In the second part of this study, we sought to overcome this limitation to vaccine effectiveness by designing and testing an inactive form of TcdB modified to adopt only the open conformation. Treatment with this constitutively open form of TcdB resulted in an effective neutralizing antibody response in all the experimental mice. Vaccination reduced weight loss, preserved colon length and reduced selected measures of inflammatory-cell infiltration. These findings provide structure-based insights into the rational design of next generation vaccines that overcome TcdB’s conformational dynamics.

## Author summary

*Clostridioides difficile* is a major cause of severe gastrointestinal infections. Much of the damage caused during infection is driven by the large multidomain toxin, TcdB, making it a worthwhile target for vaccine and therapeutic development. However, TcdB exhibits extensive conformational flexibility, transitioning between open and closed forms, and the impact of these structural changes on the neutralizing ability of vaccine induced antibodies is not well understood. Here, we evaluated the immune response and protective ability of a detoxified TcdB vaccine candidate against infection. Despite eliciting a strong antibody response, vaccination provided only limited protection against infection. We further found these vaccine-induced antibodies poorly neutralized a TcdB mutant unable to adopt the closed conformation. In contrast, immunization with this conformationally restricted toxin generated broadly neutralizing antibodies and improved protection against inflammatory-cell infiltration. Our data raise the possibility that TcdB conformational flexibility could be a potential immune evasion strategy that could diminish antibody mediated protection and vaccine efficacy.

## Introduction

The bacterial toxin TcdB is produced by *Clostridioides difficile* during infection and is an indispensable virulence factor necessary for disease [1–4]. As a 270 kDa intracellularly acting toxin, TcdB binds cell surface receptors, undergoes autoprocessing, and translocates into the cytosol of cells where it inactivates small GTPases, effectively destroying or incapacitating target cells [5–10]. Recent studies show that TcdB, and the related toxin TcdA, adopt multiple conformational states affected by environmental conditions such as pH [11–14]. These studies reveal two distinct conformational states in which TcdA and TcdB adopt structures wherein the carboxy terminal region, covering approximately a fourth of the protein, undergoes a dramatic reorientation that renders the toxins in either an open or closed conformation. Proposed models indicate the closed conformation of TcdB is unable to intoxicate cells and has a particular susceptibility to inactivation by bile-salts and antibodies [13,15,16]. Conversely, the open conformation is capable of receptor binding and, purportedly, membrane insertion necessary for translocation into the cytosol [13,17–19].

Many of the clinical manifestations of *C. difficile* infection (CDI) are attributable to TcdB and isogenic strains lacking this toxin are avirulent, making TcdB a leading candidate for therapeutic interventions and vaccination [1,2,20,21]. Yet, results from two phase 3 clinical trials using vaccines that included TcdB failed to yield sufficient protection against disease [22–25]. These setbacks occurred with seemingly strong vaccine candidates and raised concerns about the efficacy of toxoid-based strategies for vaccinating against CDI [26]. Although the reasons for the limited success of TcdB-related vaccines are poorly understood, the toxin’s conformational dynamics could impact its antigenic profile while also providing a mechanism to escape antibody-mediated neutralization.

Previous studies suggest TcdB conformational variations could hinder antibody interactions with neutralizing epitopes. Studies comparing antibody reactivity between TcdB1 and TcdB2 found that conformational dynamics altered epitope accessibility in TcdB2 [27] and this was directly influenced by a 99 amino acid region proximal to the reactive epitopes [28]. Deletion of or altering sequences in the 99 amino acid region (residues 1753–1851) led to broader epitope exposure [28,29]. Further analysis narrowed this to a 19 amino acid subdomain (residues 1769–1787) that, coincidentally, was also found to be necessary for TcdB cell entry [30]. This region is also structurally adjacent to a key hinge sequence that reorganizes when the carboxy terminal combined repetitive oligopeptides (CROPs) domain adopts either the open or closed conformation [12,13]. These observations supported the development of TcdB2_Δ1769–1787_ as a vaccine candidate with reduced toxicity and that could potentially generate broader neutralizing antibodies by eliminating structural restraints on epitope exposure [31]. To optimize this vaccine, work in the current study further modified TcdB2_Δ1769–1787_ to eliminate both autoprocessing and glucosyltransferase activity. The new vaccine candidate (termed Vac-1) was subjected to detailed measurements related to humoral immune responses and protection from *C. difficile* infection.

In parallel with testing a conformationally-modified form of TcdB as a vaccine candidate, we also explored how the open and closed conformations might alter antibody-mediated neutralization following vaccination. While the previous studies provide strong evidence that structural differences between TcdB variants influence epitope accessibility, they do not account for the intrinsic dynamic nature of the toxin. Through experimental comparisons using full-length TcdB and a constitutively open form of TcdB, we measured the effects of conformational states on vaccine-induced antibody reactivity to the toxin. Our results indicate that distinct conformational states (e.g., open or closed) have a profound impact on vaccine effectiveness and raises the possibility that TcdB’s structural dynamics may have evolved, at least in part, to limit recognition by neutralizing antibodies

## Results

### Rationale and design of vaccine candidate Vac-1

**Fig 1:**
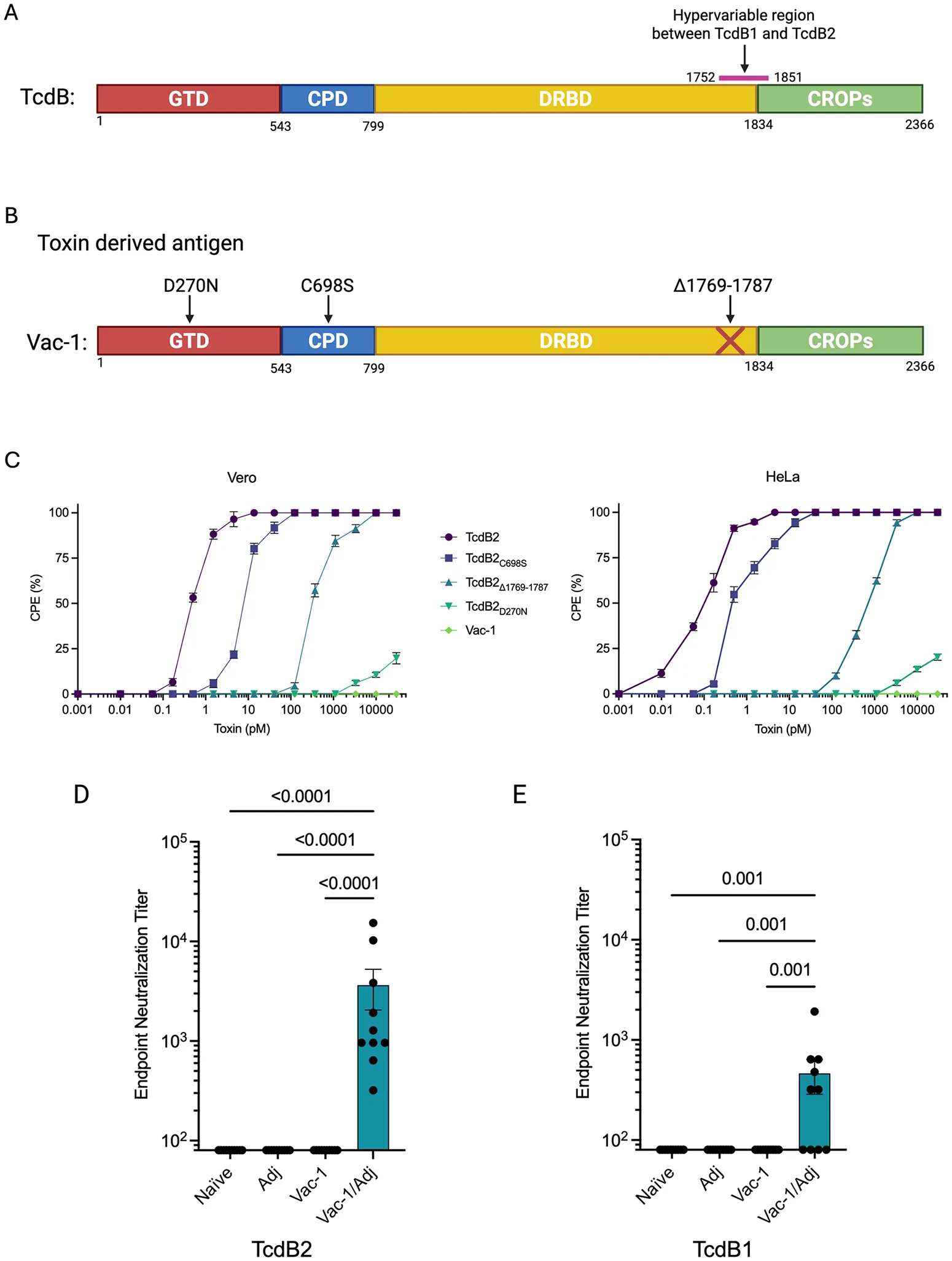
Design and safety of the Vac-1 vaccine candidate and its ability to elicit neutralizing antibodies in mice. (A) Schematic of TcdB showing the four functional domains (GTD: glucosyltransferase domain, CPD: cysteine protease domain, DRBD: delivery/receptor binding domain, CROPs: carboxy-terminal domain containing combined repetitive oligopeptide sequences). Numbers refer to amino acid residue number. The hypervariable region between TcdB1 and TcdB2 is highlighted in pink. (B) Schematic of Vac-1 depicting three mutations compared to TcdB2 (D270N, C698S, and Δ1769-1787). (C) Cytotoxicity assays quantifying cytopathic effects (CPE) of recombinantly produced TcdB2 and associated mutants on Vero and HeLa cells. The means of four biological replicates ± SD are shown. The ability of mouse sera to neutralize (D) TcdB2 and (E) TcdB1 was measured by incubating serial dilutions of sera with a constant concentration of toxin before application to Vero cells. The inverse of the minimum dilution that protected 50% cell rounding is plotted. Duplicate values are depicted for samples analyzed independently in different plates. Each symbol represents an individual mouse, and bars denote the mean of each group ± SEM. Statistical significance was determined using a Kruskal-Wallis test followed by Dunn’s multiple-comparison test. Statistically significant *P* values are shown.

Previous work found that TcdB2_Δ1769–1787_ generates neutralizing antibody responses to TcdB2 and protected against disease in a *C. difficile* mouse infection model [31]. We sought to improve upon this vaccine candidate by introducing two additional mutations (Fig 1A–B) to ensure complete detoxification. A D270N mutation was introduced, which removes the aspartate residue necessary for hydrolysis of UDP-glucose and thus glucosylation of small-GTPases [32,33]. Second, given TcdB2_Δ1769–1787_ demonstrated spontaneous autoprocessing, a C698S mutation was introduced to inactivate the cysteine protease activity of TcdB2. The TcdB2_D270N:C698S:Δ1769–1787_ mutant was renamed Vac-1 and was recombinantly expressed as a His-tagged protein in *Bacillus megaterium* and purified. To confirm full detoxification of TcdB, the cytotoxicity of Vac-1 was compared to individual mutants consisting of TcdB2_D270N,_ TcdB2_C698S_, and TcdB2_Δ1769–1787_. Although the single point and deletion mutants exhibited reduced but detectable cytotoxicity, treatment of cells with Vac-1 did not result in any detectable cell rounding or cell lysis at concentrations as high as 30 nM (Fig 1C).

### Vac-1 stimulates a robust humoral immune response

To determine the immunogenicity of Vac-1 and its ability to induce an effective IgG response, mice were vaccinated with Vac-1, Alhydrogel adjuvant-adsorbed Vac-1 (Vac-1/adj), an equal volume of Alhydrogel only or PBS as a vehicle control (S1A Fig). After 28 days, mice received a booster dose of Vac-1. Blood was collected on day 28 prior to receiving a booster dose and on day 35 and serum antigen-specific IgG antibody titers were measured by ELISAs (S1B-E Fig and S2A-D Fig). Immunization with Vac-1/adj induced a significant primary and recall IgG1 response compared to naïve, adjuvant only, and Vac-1 only controls (S1B Fig and S2A Fig). Mice that received Vac-1/adj also demonstrated higher titers of IgG2b compared to the other groups (S1C Fig and S2B Fig). Additionally, there were similar IgG1 and IgG2b titers when using TcdB2 as the ELISA coating antigen compared to Vac-1 as the antigen (S1D-E Fig and S2C-D Fig), confirming the antibodies made in response to Vac-1 also bind the wildtype (WT) TcdB2 toxin.

Previous work has indicated that antibodies capable of neutralizing TcdB can protect against disease [20,34–37]. To determine whether antibodies generated in response to Vac-1 could neutralize toxin, the ability to reduce the cytopathic effects of both TcdB2 and TcdB1 was measured. Sera samples were serially diluted and incubated with recombinantly purified TcdB2 or TcdB1 before application to Vero cells. The inverse of the dilution conferring 50% protection against cell rounding was plotted (Fig 1D–E and S2E-F Fig). Only sera from mice immunized with Vac-1/adj neutralized TcdB2 (Fig 1D and S2E Fig). Higher serum concentrations were required to neutralize TcdB1 than TcdB2, and only a subset of mice produced sera capable of neutralizing TcdB1 (Fig 1E and S2F Fig).

While most plasma cells generated immediately after boosting are short lived and found in circulation, the cells detected in the bone marrow represent those that have successfully migrated and have the potential to contribute to long-term humoral immunity. To assess the plasma cells that had begun to establish in the bone marrow, IgG1 secreting cells were enumerated from bone marrow leukocytes using an enzyme-linked immunosorbent spot assay (ELISPOT) 14 days post-boost (S1F Fig). Mice that received Vac-1/adj had an abundance of IgG1 secreting cells specific for the antigen while the control mice did not (S1 G-H Fig).

The formation of germinal centers (GCs), specialized microenvironments in lymphoid organs where B cells mature to produce high-affinity antibodies, is a critical step in the adaptive immune response. Mice were immunized with Vac-1/adj or PBS and after 14 days the draining inguinal lymph nodes (iLNs), were collected, sectioned, stained with hematoxylin and eosin (H&E) and visualized (S3A-B Fig). Vac-1/adj induced the formation of significantly more GCs in the iLNs compared to the naïve controls (S3C Fig). Additionally, there was no difference in the number of B cell follicles present in the iLNs, the region where GCs form in the lymph nodes, between the two groups, but the mice receiving Vac-1 had a greater percentage of follicles that contained GCs (S3D-E Fig).

To evaluate whether Vac-1 stimulated B cell activation, we quantified memory B cells (Bmems) from the iLNs (S3F-I Fig). The frequency and absolute number of Bmem cells was determined from the iLNs collected from mice 60 days post immunization with either Vac-1/adj or vehicle control, using flow cytometry (S3F-I Fig). The frequency of antigen-activated B cells (IgD^-^/CD19^+^) was significantly higher in mice that received Vac-1/adj than the naïve mice (S3H Fig). Additionally, the average absolute number of Bmem cells per iLN in the mice that received Vac-1/adj was significantly higher than the group that received the vehicle control (S3I Fig).

### Vac-1 reduces weight loss in a mouse model of CDI

**Fig 2:**
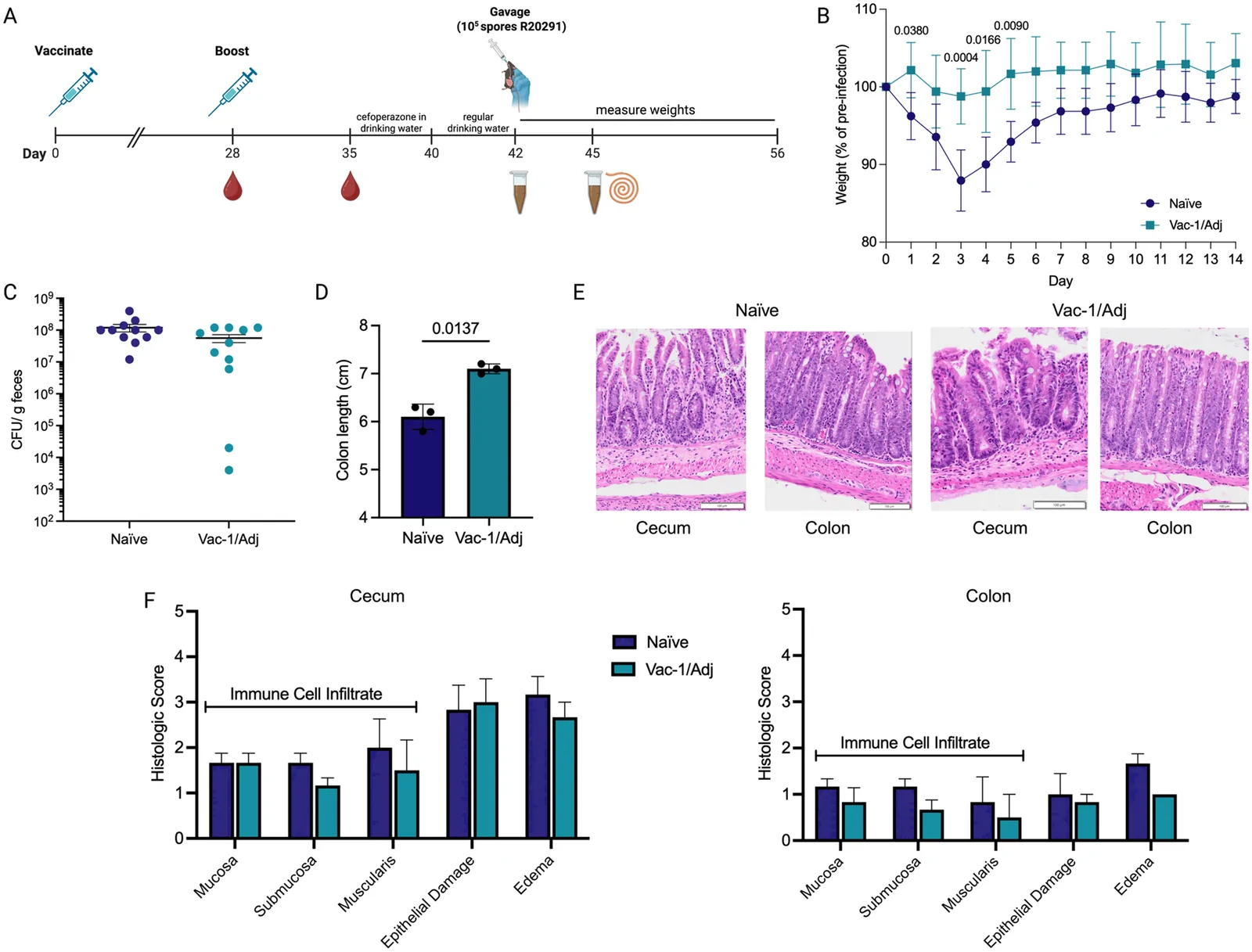
Effect of Vac-1 on severity of CDI in a mouse model. (A) Mice received a primary dose of Vac-1/adj (n = 12) or an equal volume of PBS (naïve; n = 12). A booster dose was given on day 28. Blood samples were collected on day 28 pre booster and day 35. Mice were given water containing Cefoperazone for 5 days starting on day 35. Mice were switched to non-antibiotic water for 2 days before being gavaged with *C.* *difficile* R20291 spores on day 42. (B) Daily weights post infection were recorded for 14 days. The mean ± SEM for each group is shown. Statistical analysis was performed using a mixed-effects model for repeated measures, with treatment group, time, and group x time interaction included as fixed effects and accounting for repeated measures from individual mice. Where the interaction term was significant Sidak’s multiple-comparison test was used to compare groups at individual timepoints. (C) Fecal pellets were collected on day 3 post gavage to enumerate *C. difficile* CFUs per gram of feces. Colonic and cecal tissues were excised from a subset of mice on day 3 post infection and (D) colon lengths were measured. Each symbol depicts an individual mouse, and bars denote the mean of each group ± SEM in C-D. Statistical significance was determined using a two-tailed Mann-Whitney U test. (E) Representative H&E images for an individual mouse in each group are depicted. (F) Histology scoring was performed (n = 3 for each group) as described in the methods section. Given the limited sample size, histological scores are presented descriptively. Data from one representative experiment are shown here; data from a second experiment are shown in Supplementary S4 Fig. Statistically significant *P* values are shown.

Given that Vac-1 was able to produce several hallmarks of an effective immune response, we next tested whether vaccination with Vac-1 was protective against CDI in a mouse model. Mice were immunized and boosted (Fig 2A) and then challenged with *C. difficile* R20291 spores. Vaccination with Vac-1/adj significantly reduced weight loss on several days post infection compared to naïve mice (Fig 2B and S4A Fig) while Vac-1 or adj alone did not (S4A Fig). There was a reduction in *C. difficile* CFUs/g of feces in mice that received Vac-1 or Vac-1/adj compared to the naïve group. This difference was significant in one experiment (S4B Fig) but not in the other (Fig 2C). Colonic and cecal tissue were prepared for histology scoring as described in the methods. Colon lengths were significantly shortened in naïve mice compared to vaccinated mice, a typical sign of severe inflammation and tissue damage (Fig 2D). Immune cell infiltration, epithelial cell damage, and edema were evaluated. Similar histological scores were observed between the naïve group and vaccinated group (Fig 2E–F). While Vac-1 immunization resulted in less weight loss during CDI, it had limited impact on overall gut pathology during CDI.

### TcdB structural dynamics impact sensitivity to antibody-mediated neutralization

**Fig 3:**
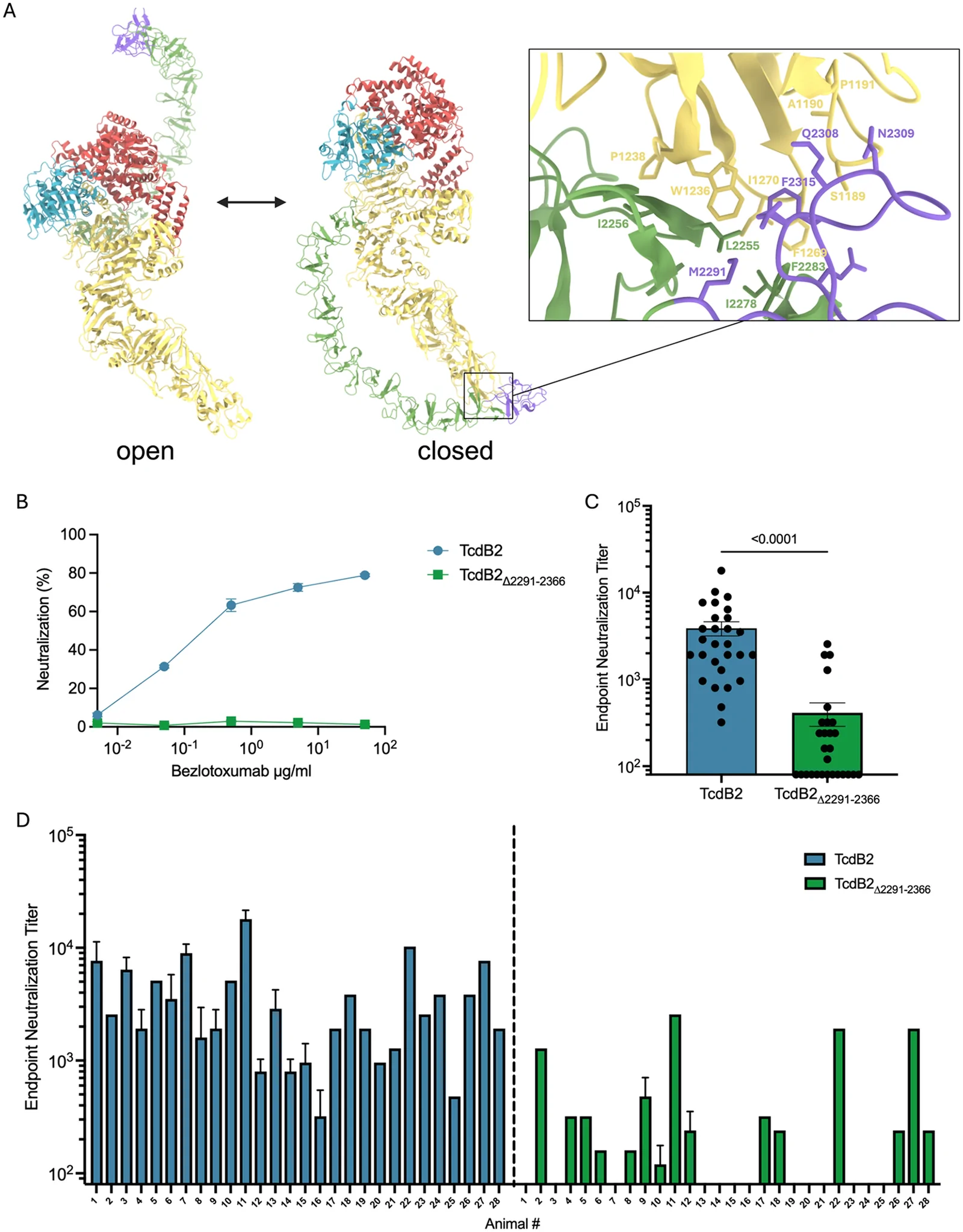
Structural conformation of TcdB affects the ability of Vac-1 sera to neutralize toxin. (A) TcdB can adopt an open (derived from PDB: 9OHF) or closed conformation (derived from PDB: 8QEN). The GTD is shown in red, the CPD in blue, the DRBD in yellow, CROPs in green, and residues 2291-2366 in purple, corresponding to the region deleted in TcdB2_Δ2291-2366_. The inset shows the predicted intramolecular interactions stabilizing the closed conformation; only residues predicted to make these contacts are labeled and color-coded by the domain to which each residue belongs. (B) TcdB2 or TcdB2_Δ2291-2366_ were incubated with different concentrations of Bezlotoxumab before being applied to CHO cells. After 24 h, cells were imaged and neutralization was measured as protection against cell rounding. (C-D) Sera collected from mice that had received a primary dose of Vac-1/adj and a booster dose 28 days later (n = 28) were tested for the ability to neutralize WT TcdB2 and TcdB2_Δ2291-2366._ Serial dilutions of sera were incubated with a constant concentration of toxin before being applied to Vero cells. The inverse of the dilution that elicited 50% protection from cell rounding is plotted. Each symbol depicts an individual mouse, and bars denote the mean of each group ± SEM in C. Statistical significance was determined using Wilcoxon signed-rank test. (D) Bars represent the mean of two technical replicates for each individual mouse.

The absence of Vac-1 induced protection against intestinal damage was unexpected given that Vac-1 elicited neutralizing antibodies that prevented TcdB cytotoxicity *in vitro*. Considering this observation, we were curious to know if TcdB might exist in conformational states that are recalcitrant to antibody neutralization. Indeed, recent studies have shown that the sensitivity of TcdB to both the FDA-approved monoclonal antibody Bezlotoxumab and bile acids is influenced by the conformational state of the CROPs domain [13,15,16]. TcdB has been reported to adopt two major conformations [12–14]. In the “closed” conformation the CROPs domain lies parallel with the delivery and receptor binding domain (DRBD) which is stabilized by multiple intramolecular contacts [13] (Fig 3A). Bezlotoxumab and certain bile acids appear to trap TcdB in the closed form thereby blocking its ability to bind to host cells [13,15,16]. In contrast, in the “open” conformation (Fig 3A) the CROP domain swings away from the DRBD, relieving hindrance to receptor engagement on host cells [13]. Therefore, we sought to determine whether antibodies generated in response to Vac-1 differed in their ability to neutralize WT TcdB2, which can adopt both structural conformations, compared to a mutant TcdB2 (TcdB2_Δ2291–2366_) predicted to remain in the open conformation [38].

TcdB2_Δ2291–2366_ is a 76-amino acid truncated mutant of TcdB2 that lacks four of the eight CROP residues predicted to form intramolecular contacts with the DRBD domain (Fig 3A) but retains WT levels of cytotoxicity [13,38]. Unlike TcdB2, TcdB2_Δ2291–2366_ was highly resistant to neutralization by Bezlotoxumab, in line with its inability to adopt a closed conformation (Fig 3B). Consistent with this, Miletic et al. demonstrated that a similar truncated C-terminal form of TcdB (TcdB_1-2100_), existed predominantly in the open conformation when examined using CryoEM [15]. Sera collected on day 35 from mice (n = 28) in the previous experiments, which had received a primary Vac-1/adj immunization followed by a booster on day 28, were tested for the ability to neutralize TcdB2_Δ2291–2366_. Sera from nearly half of the mice (13/28) failed to neutralize TcdB2_Δ2291–2366_ and among those that did (15/28), much higher sera concentrations were required to do so compared to WT TcdB2 (Fig 3C–D). These findings show that the ability of different antibodies to neutralize TcdB is influenced by the conformational dynamics of the toxin.

### Vaccination with TcdB2_D270N_Δ2291–2366_ reduces weight loss in a CDI mouse model and induces the production of TcdB2_Δ2291–2366_ neutralizing antibodies

**Fig 4:**
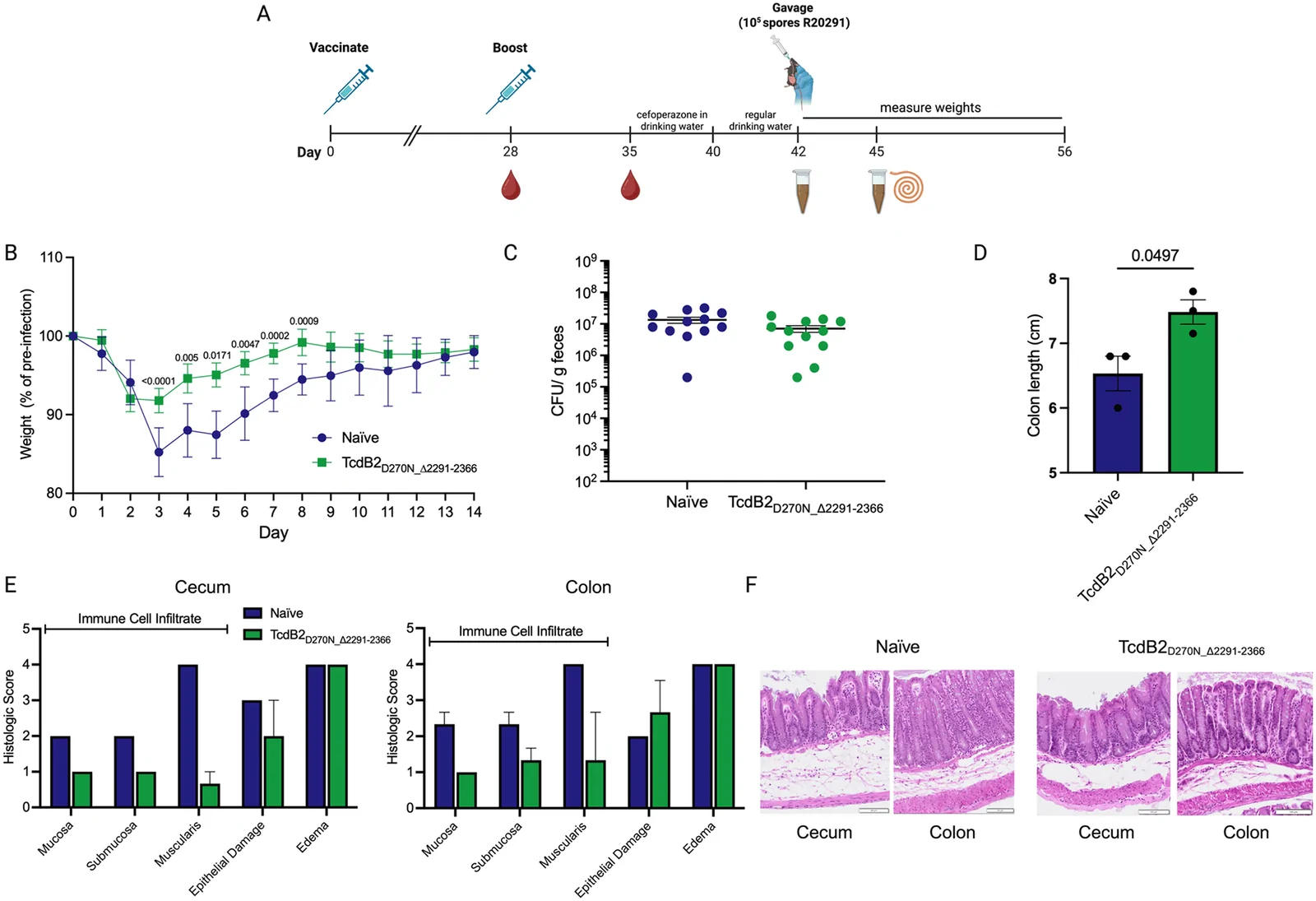
Effect of TcdB2_D270N___Δ__2291-2366_ on severity of CDI in a mouse model. (A) Mice received a primary dose of TcdB2_D270N___Δ2291-2366_/adj (n = 12) or an equal volume of PBS (naïve; n = 12). A booster dose was given on day 28. Blood samples were collected on day 28 pre booster and day 35. Mice were given water containing Cefoperazone for 5 days starting on day 35. Mice were switched to non-antibiotic water for 2 days before being gavaged with *C. difficile* R20291 spores on day 42. (B) Daily weights post infection were recorded for 14 days. The mean ± SEM for each group is shown. Statistical analysis was performed using a mixed-effects model for repeated measures, with treatment group, time, and group x time interaction included as fixed effects and accounting for repeated measures from individual mice. Where the interaction term was significant Sidak’s multiple-comparison test was used to compare groups at individual timepoints. (C) Fecal pellets were collected before the gavage and on day 3 post gavage to enumerate *C. difficile* CFUs per gram of feces. Colonic and cecal tissues were excised from a subset of mice on day 3 post infection and (D) colon lengths were measured. Each symbol depicts an individual mouse, and bars denote the mean of each group ± SEM. Statistical significance was determined using a two-tailed Mann-Whitney U test. (E) Histology scoring was performed (n = 3 for each group) as described in the methods section. Given the limited sample size, histological scores are presented descriptively. (F) Representative H&E images for an individual mouse from each group are depicted. Statistically significant *P* values are shown.

To further interrogate how TcdB conformational dynamics influence the production of neutralizing antibodies, mice were immunized with the putatively open truncated TcdB2. A D270N alteration was introduced to the TcdB2_Δ2291–2366_ mutant to remove glucosyltransferase activity. Mice were immunized with either PBS or TcdB2_D270N_Δ2291–2366_/adj as previously described, boosted on day 28, and then challenged with *C. difficile* R20291 spores on day 42 (Fig 4A). Mice that received TcdB2_D270N_Δ2291–2366_/adj lost significantly less weight than naïve mice starting 3 days post infection and continuing until day 8 (Fig 4B). No difference in CFUs per gram of feces was observed indicating no change in overall *C. difficile* colonization between the two groups (Fig 4C). Colonic and cecal tissues were excised from a subset of mice on day 3 post infection. Colon lengths were significantly shortened in naïve mice (Fig 4D) indicating more severe tissue inflammation than the mice that had received the vaccine. Immune cell infiltrate was also reduced in mice that received TcdB2_D270N_Δ2291–2366_/adj compared to naïve mice, with the largest reduction observed in the muscularis of the cecum (Fig 4E and F).

**Fig 5:**
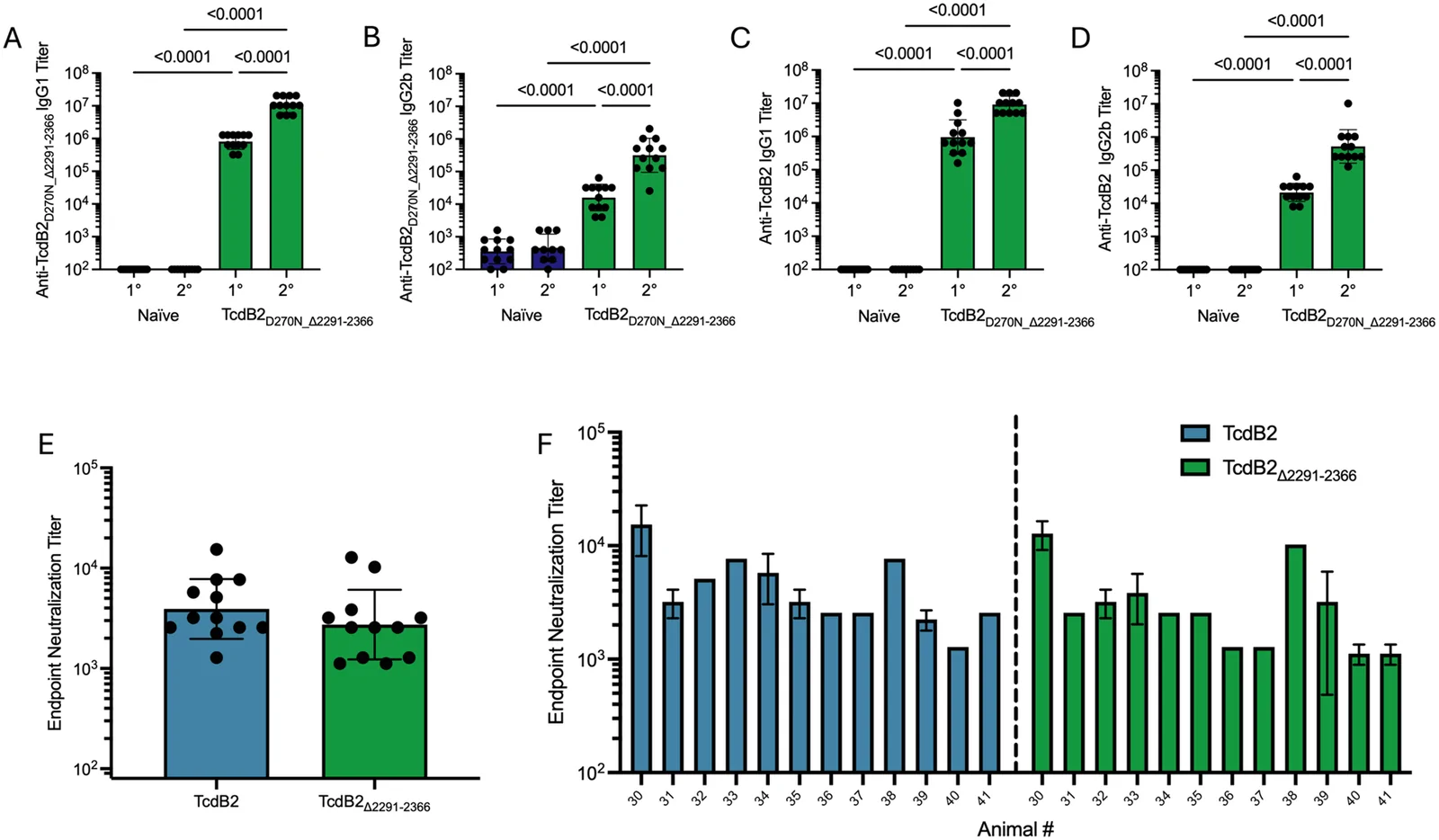
TcdB2_D270N___Δ__2291-2366_ induces robust IgG production and broadly neutralizing antibodies. Primary (1°) and recall (2°) IgG1 and IgG2b sera titers were measured using ELISAs with (A-B) TcdB2_D270N___Δ2291-2366_ as the coating antigen or (C-D) TcdB2 as the coating antigen. Bars show geometric mean titers ± SD per group with each symbol representing an individual mouse. (A-D) Significance was determined by a two-way ANOVA with Sidak’s multiple comparison post-test. (E-F) The ability of mouse sera to neutralize TcdB2 and TcdB2_Δ2291-2366_ was measured by incubating serial dilutions of sera with a constant concentration of toxin before application to Vero cells. The inverse of the minimum dilution that protected 50% cell rounding is plotted. Duplicate values are depicted for samples analyzed independently in different plates. (E) The mean ± SEM is depicted for each group with each symbol representing an individual mouse. Statistical significance was determined using Wilcoxon signed-rank test. (F) Bars represent the mean ± SEM of two technical replicates for each individual mouse. Statistically significant *P* values are shown.

Serum antigen-specific IgG antibody titers were measured from blood collected on day 28 prior to receiving a booster dose and on day 35 using ELISAs. Immunization with TcdB2_D270N_Δ2291–2366_/adj induced a significant primary and recall IgG1 and IgG2b response compared to the naïve group (Fig 5A–D). Additionally, there were similar IgG1 and IgG2b titers when using TcdB2 as the antigen (Fig 5C–D), confirming the antibodies made in response to TcdB2_D270N_Δ2291–2366_ also bind WT toxin. We next assessed the functional capacity of the vaccine elicited antibodies to neutralize toxin. Notably, the antibodies produced in response to TcdB2_D270N_Δ2291–2366_ were equally capable of neutralizing WT TcdB2 and the “open” TcdB2_Δ2291–2366_ (Fig 5E–F), suggesting these antibodies broadly neutralize distinct conformational states of TcdB and that the structural conformation of the toxin can greatly influence the exposure of certain neutralizing epitopes thereby shaping the antibody response.

## Discussion

TcdB neutralizing antibodies reduce *C. difficile* disease severity and recurrence and is an important correlate of protection against CDI [20,36]. Accordingly, TcdB based vaccines represent a promising therapeutic strategy. Despite this, several toxin-based vaccines that elicited strong neutralizing antibody responses have failed in clinical trials [22–25,39]. Results from the current study, indicate TcdB’s conformational dynamics could contribute to limited effectiveness of vaccines that target this critical *C. difficile* virulence factor. Importantly, the data suggest this obstacle to effective TcdB-based vaccination could be overcome by driving immunity solely to the open and fully cytotoxic form of this toxin.

In this study, we eliminated residual toxicities in vaccine candidate TcdB2_Δ1769–1787_ by introducing two additional mutations (D270N and C698S) that removed glucosyltransferase and autoprocessing activities, respectively. Even at high concentrations, we were unable to detect any cytotoxic effects from Vac-1 (Fig 1). Vaccination with Vac-1/adj elicited a robust humoral immune response characterized by the production of high IgG titers that recognized both Vac-1 and TcdB2, indicating the modifications in Vac-1 preserved reactive epitopes present in the holotoxin (S1 Fig and S2 Fig). Vac-1 also promoted B cell activation and differentiation, as evidenced by germinal center formation, expansion of Bmems, and the generation of antibody secreting cells in the bone marrow (S1-3 Figs). Additionally, mice that received Vac-1/adj demonstrated reduced weight loss compared to naïve mice in a CDI model (Fig 2). Curiously, Vac-1 failed to protect against colonic and cecal tissue damage despite the apparent strong neutralizing effect measured *in vitro*.

We wanted to better understand why mice exhibited intestinal damage in the presence of neutralizing antibodies against TcdB. Results from several prior studies led us to conclude that the damage was unlikely to be due to TcdA, a second *C. difficile* toxin related to TcdB. A collection of previous reports indicates that in the absence of TcdB (e.g., a *tcdB* deletion strain), pathologies are limited and TcdA alone cannot account for intestinal damage [1–3,40]. This led us to consider whether TcdB itself might avoid complete neutralization by fluctuating between antibody-sensitive and antibody-resistant conformations. To further investigate this possibility, we examined whether the neutralizing abilities of Vac-1 generated antibodies were influenced by the two known structural conformations of TcdB. The results shown in Fig 3 indicated that sera from Vac-1 immunized mice effectively neutralized WT TcdB2, which can sample both the open and closed conformations, but exhibited markedly reduced neutralization to TcdB2_Δ2291–2366_, a mutant unlikely able to form the closed conformation. These data suggest that these antibodies target epitopes inaccessible when the toxin is restricted to the open state and thus only likely neutralize a subpopulation of WT TcdB2.

To directly test whether conformational states could influence vaccination, we generated a mutant of TcdB2_Δ2291–2366_ (open conformation) that also lacked glucosyltransferase activity and examined its effectiveness as an immunizing antigen. Similar to Vac-1, mice that received TcdB2_D270N_Δ2291–2366_ demonstrated a strong anti-TcdB2 IgG response accompanied by reduced weight loss post infection with *C. difficile* (Fig 4–5). While similar levels of edema and epithelial damage were observed in both cecum and colon samples between the naïve group and vaccinated group, less overall immune cell infiltrate was observed in the group that had received TcdB2_D270N_Δ2291–2366_ suggesting some protection against disease progression. Interestingly, the antibodies produced in response to TcdB2_D270N_Δ2291–2366_ were equally capable of neutralizing WT TcdB2 and the “open” mutant suggesting the exposed epitopes in this structurally restricted TcdB2 may be more broadly neutralizing than those exposed in Vac-1 (Fig 5).

There are many examples of bacterial toxins that require activation, typically through proteolytic cleavage or engaging co-factors, to elicit cytotoxic effects. Though TcdB has not been classified as such, it is notable that at neutral pH the toxin exists in a dynamic equilibrium between open and closed conformations rather than predominantly adopting the receptor accessible state [12,13].This raises important questions about what advantage TcdB gains by adopting a conformation that is non-toxic and can be exploited by bile salts for inactivation within the intestines [15,16,41]. TcdB’s closed conformation has been proposed to prevent premature autoprocessing [12,19,42,43], but structural studies show that the open conformation is necessary for receptor binding [13,18,19]. As such, to bind receptor and initiate cell entry TcdB should adopt an open conformation before autoprocessing would occur within the cell. Moreover, TcdB samples either open or closed structures stochastically at neutral pH, which should result in autoprocessing but TcdB does not spontaneously autoprocess in solution. Thus, TcdB’s necessity for the closed conformation is unlikely to be driven solely by the need to prevent premature autoprocessing. Data from the current study suggest a new and intriguing hypothesis: TcdB’s conformational dynamics misdirect antibody responses to forms of the toxin that are not susceptible to neutralization. TcdB’s vacillation between the open and closed conformation could provide a mixed population of toxin that exhibits antibody susceptible and antibody resistant forms of the protein, representing a sophisticated mechanism of escaping complete neutralization and preserving cytotoxicity during infection. In line with this, it is notable that previous studies examining antibody repertoires in recovered *C. difficile* patients found that a vast majority of TcdB-reactive antibodies are unable to neutralize the toxin [44].

Our findings complement recent advances in CDI vaccine development reported by two other groups, which addressed important aspects of vaccine design beyond the toxin antigen itself. Alameh et al. developed a multivalent mRNA–LNP vaccine targeting both TcdA and TcdB as well as additional *C. difficile* antigens, demonstrating protection against severe disease and mortality and highlighting the potential of combining toxin- and pathogen-directed immunity [45]. Thomas et al. subsequently demonstrated that the route and localization of the immune response are critical determinants of vaccine efficacy [46]. Their multivalent mucosal vaccine incorporated inactivated toxins together with surface and spore-associated antigens and, when administered rectally, induced mucosal immune responses that promoted bacterial clearance and protected against morbidity, tissue damage, and recurrence. In contrast to these approaches, our study focuses specifically on optimizing the toxin antigen itself. We demonstrated that TcdB’s conformational states impact both effectiveness of the vaccine itself and the toxin’s ability to avoid neutralizing antibodies. Accounting for these TcdB characteristics should be a consideration in both vaccine design and measuring vaccine efficacy when TcdB is used a single antigen or in combination with multiple antigens to vaccinate against CDI.

Future work focused on defining the regions of TcdB recognized by Vac-1 versus TcdB2_D270N_Δ2291–2366_ elicited antibodies will help determine which epitopes might be conformation-sensitive and may help guide the design of improved vaccine candidates. Further, using conformationally restricted variants of TcdB that force the immune system to focus on a single conformation appears to be a promising avenue for vaccinating against this critical *C. difficile* virulence factor.

## Materials and methods

### Ethics statement

All animal procedures were approved by the Institutional Animal Care and Use Committee at the University of Oklahoma Health Science Center (protocol 23–037-ACHIU). This study was performed in accordance with the guidelines set forth in the Guide for the Care and Use of Laboratory Animals of the National Institutes of Health.

### Construction and purification of recombinant toxins

The nucleotide sequence of the vector pC-His1622-*tcdB2* [28] was modified to result in three mutations at the amino acid level: D270N, C698S, and Δ1769–1787 by GenScript (Piscataway, NJ). The resulting vectors were transformed into *Bacillus megaterium* WH320 (MoBiTec) following the manufacturer’s guidelines. Recombinant toxins were affinity purified using nickel chromatography as described previously [28]. Bacterial strains and plasmids used in this study can be found in S1 Table.

### Cytotoxicity assays

All cell lines were purchased from the American Type Culture Collection (ATCC) and cultured at 37°C with 5% CO_2._ Vero-E6 cells were grown in Dulbecco’s Modified Eagle Medium (DMEM) containing 5% fetal bovine serum (FBS), 100 units/ml penicillin, and 100 µg/ml streptomycin. HeLa cells (CCL-2) were cultured in Eagle’s Minimum Essential Medium supplemented (EMEM) with 10% FBS, 100 units/ml penicillin, and 100 µg/ml streptomycin. CHO K-1 cells were cultured in F-12-K medium containing 10% FBS, 100 units/ml penicillin, and 100 µg/ml streptomycin. Cells were seeded in a 96-well plate at a density of 10,000 cells per well and grown overnight. The next day, cells were exposed to various concentrations of toxins as indicated. The following day cells were imaged and rounded and non-rounded cells were manually counted.

### Vac-1 and TcdB2_D270N_Δ2291–2366_ Immunizations

Six-week-old female C57Bl/6 mice were purchased from Jackson Laboratories. We chose to use only female mice to maintain consistency with our previous work. In our experience with the *C. difficile* mouse model, we have not observed apparent sex-related differences in disease severity or experimental outcomes [47]. Mice were immunized with a 200 µl subcutaneous injection divided between both rear flanks. Injections consisted of 50 µg of the designated antigen in sterile PBS. Antigen/adj indicates adsorption to a 2% (w/v) Alhydrogel alum suspension (Invivogen). Where indicated, mice received a booster dose (25 µg) on day 28. Control mice received an equal volume of PBS or adj. Retro-orbital blood collections were conducted on day 28 (pre booster) and day 35 using heparin capillary tubes. Blood samples were incubated for 2 h at room temperature (RT), centrifuged at 13,000 x g for 15 min. Sera were collected and stored at -20°C.

### ELISAs

Nunc MaxiSorp 96 well plates were coated overnight at 4°C with phosphate coating buffer (0.1 M Na_2_HPO_4_, pH 9.0) containing the indicated antigen at a final concentration of 10 µg/ml. Each well was washed 4 times with PBS-T (1X PBS, 0.05% Tween) and blocked with 1% BSA in PBS-T for 2 h at RT. Wells were washed 4 times again with PBS-T and then incubated with mouse sera (serially diluted 2-fold) overnight at 4°C. The next day, wells were washed 4X again and then incubated with 0.2 µg/ml HRP-conjugated IgG1 or IgG2b for 1 h at 4°C. Wells were washed with PBS-T and developed for 5 min at RT by addition of 90 µl of 2,2′ azinobis(3-ethylbenzthiazolinesulfonic acid) (ABTS) substrate to each well (SeraCare). 110 µl of 10% w/v SDS was added to stop the reaction. Optical density (OD_405 nm_) of each well was measured within 30 min. Endpoint ELISA titers were determined by two-fold serial dilution of sera until absorbance values were 0.02 or less and could not be distinguished from background.

### Neutralization assays

Vero or CHO cells were cultured and seeded as described above. The concentration of each toxin that resulted in ~95% cell rounding (EC95) was determined and kept constant in these assays (1.6 ng/ml TcdB2 and 0.8 ng/ml TcdB2_Δ2291–2366_ for Vero cells; 0.2 ng/ml for both TcdB2 and TcdB2_Δ2291–2366_ for CHO cells). Serial dilutions of sera or the indicated concentration of Bezlotoxumab (synthesized by GenScript) were mixed with the indicated toxin in complete culture media and incubated for 30 min at 37°C and then applied to cells. Wells without any toxin or only toxin were included as controls. The cells were incubated overnight at 37°C and imaged the next day. The inverse of the minimum dilution that protected 50% cell rounding was plotted.

### Germinal center analysis

Mice were immunized as described above with Vac-1/adj or PBS. On day 14 post immunization the mice were euthanized, and one iLN was collected per mouse and placed in Modified Davidson’s fixative. Paraffin sections (5 µm thick) were mounted on slides by Excalibur Pathology Inc. (Norman, OK), with at least 15 sections per slide. Sections were stained with H&E and mounted with a coverslip. Slides were imaged on a Zeiss Digital Slide Scanner Axioscan (Hebron, KY) at 20X. Germinal centers and follicles were enumerated manually. Germinal center area was measured on the section that contained the largest cross section and was calculated automatically using the circle area tool. Each germinal center was measured once. All image analysis was done using the Zen Blue software (Zeiss).

### ELISPOTS

Mice were vaccinated and boosted as described above. Mice were euthanized on day 42, leg bones were collected, and bone marrow (BM) was harvested [48,49]. Bone marrow cells were flushed from the tibia and femur with media. Splenocytes and bone marrow cells were then re-suspended gently using a 1 mL pipette. Erythrocytes were removed by incubation with ammonium chloride lysis buffer (0.16 M NH_4_Cl, 0.17 M Tris-HCl, pH 7.4) for 2 min at 37°C. After washing in culture media, cell viability was confirmed as >98% by trypan blue exclusion.

Multiscreen High-throughput Satellite (HTS) enzyme-linked immunosorbent spot (ELISPOT) wells (Millipore) were prepared for antigen coating by incubating with 35% v/v ethanol for 30 s and washed twice with PBS. The plates were coated overnight with anti-mouse Ig or Vac-1 (10 μg/mL final concentration) at 4°C. Plates were washed 3 times with PBS and blocked with RPMI 1640 containing 10% FBS for 2 h at room temperature. Isolated bone marrow cells (3 × 10^6^ cells per well) were added then subjected to a 1:3 serial dilution such that the wells contained 2.00 × 10^6^, 6.67 × 10^5^, 2.22 × 10^5^, or 7.41 × 10^4^ cells. The plates were then incubated in 5% CO_2_ at 37°C for 4.5 h. The cells were lysed and removed by 4 washes with PBS-T. Plates were incubated overnight at 4°C with 5% v/v FBS in PBS containing HRP-goat anti-mouse IgG1 and IgG2b mAb at a final concentration of 1.0 μg/mL (Southern Biotech). The plates were then washed with PBS-T, then PBS, and colorimetric detection performed. The developing solution was prepared by dissolving one tablet of 3-amino-9-ethyl-carbazole (AEC) (Sigma) in 2.5 mL dimethylformamide (Sigma) and mixing with 47.5 mL of a 0.0075 N acetic acid and 0.0175 M sodium acetate buffer. The solution was passed through a 0.2 μm syringe filter before adding hydrogen peroxide to a final concentration of 0.0005% v/v. Developing solution (100 µl) was added to each well and incubated at room temperature for 10 min. The reaction was stopped by addition of deionized water. The plates were washed 20 times and allowed to air dry at room temperature under light-protected conditions for 24 h. Antibody secreting cells (spots) were enumerated using an ImmunoSpot analyzer (Cellular Technology Limited). A positive ELISPOT response was regarded as more than 3 standard deviations above background in the assay. In this study, the background was zero and 1 or more spots were designated as a positive response.

### Detection of memory B cells by flow cytometry

Mice were vaccinated as described above and after 60 days were euthanized and inguinal and axillary lymph nodes harvested. Single cell suspensions were prepared by mechanical disruption and stained with an antibody cocktail consisting of unlabeled FcR-blocking mAb 2.4G2 (BioXCell); PE-anti-mouse B220 mAb (Clone RA3-6B2, Tonbo Biosciences); APC-anti-mouse-CD19 (Clone ID3, Tonbo Biosciences); and FITC-anti-mouse-IgD (11-26c.2a, BD Pharmingen). Cells were stained for 45 minutes at 4°C before washing three times in cold PBS. Cells were then fixed with 1% w/v paraformaldehyde in PBS. Samples were analyzed using a Stratedigm S1200Ex Flow Cytometer (San Jose, CA). Data were analyzed using FlowJo Software (Tree Star, Ashland, OR).

### *C. difficile* spore preparation

*C. difficile* spores were prepared as previously described [50,51]. A single colony grown on Brain Heart Infusion (BHI) + Taurocholic acid (TCA) agar was used to inoculate 2 ml of Columbia Broth and grown anaerobically overnight at 37°C in a Coy anaerobic chamber. The next day, the entire 2 ml culture was used to inoculate 40 ml of Clospore media. The culture was grown anaerobically at 37°C for 5–7 days. Spores were collected by centrifugation and washed with cold water at least three times until the supernatant was clear. Finally, the spore pellet was resuspended in 1 ml sterile water and stored at 4°C. Before infection, *C. difficile* spores were enumerated by plating on TCCFA (Taurocholate, Cycloserine, Cefoxitin, and Fructose Agar). The spore inoculum was heated at 65°C for 20 min, then allowed to cool for 5 min at room temperature before gavaging mice.

### *C. difficile* mouse infection model

Mice were vaccinated as described above and when indicated given distilled water containing Cefoperazone (0.5 g/l, Sigma) for 5 days. Antibiotic water was refreshed every two days. Mice were then switched to distilled water for the following two days. Mice were gavaged with 1x10^5^ heat-activated *C. difficile* R20291 spores. Clinical signs of CDI were assessed by monitoring weight, lethargy, hunched posture, and diarrhea. Animals were euthanized if they lost more than 20% of their starting weight or were moribund. *C. difficile* CFUs were enumerated on day 0 (to ensure mice were not pre colonized with *C. difficile*) and on day 3 post gavage, which represents peak infection, by plating serial dilutions of homogenized fecal pellets in PBS onto TCCFA agar. Plates were anaerobically incubated at 37°C and CFUs counted the next day. A subset of mice was euthanized on day 3 post gavage for histology. The cecum to colon was excised, separated, and the feces removed. The colon was cut lengthwise and rolled from the anal junction end. The cecum was left intact and rolled from the closed end to the intestinal junction. Once rolled, the tissues were pierced with a 30-gauge needle, placed in tissue cassettes, and fixed in Modified Davidson’s fixative (64% DI water, 20% 100% ethanol, 6% glacial acetic acid, 10% formaldehyde). Paraffin sections (5 µm thick) were mounted on slides by Excalibur Pathology Inc. (Norman, OK). Slides were stained with H&E to assess tissue damage. Slides were imaged on an Olympus SlideView VS200 at 20X using the Default Brightfield Scan protocol. Images were uploaded to HistoWiz (Long Island City, NY) for blinded disease index measurement by a board-certified veterinary pathologist using a prespecified previously published rubric by scoring immune cell infiltration, epithelial damage, and edema on a scale of 0 – 4 [52,53].

### TcdB structure visualization

The structures of the open TcdB conformation (PDB ID: 9OHF [15]) and the closed conformation (PDB ID: 8QEN [13]) were obtained from the Protein Data Bank. Images were generated using ChimeraX-1.11.1 [54].

### Statistics

Data were analyzed using GraphPad Prism V 10.1 (GraphPad Software) as described in figure legends. Only *P* values ≤ 0.05 were considered statistically significant and are shown in figures.

## Supporting information

S1 Fig Humoral immune responses elicited by Vac-1 in mice. (TIF)

S2 Fig IgG recall response and generation of neutralizing antibodies in mice vaccinated with Vac-1. (TIF)

S3 Fig Assessment of the ability of Vac-1 to induce the formation of germinal centers (GCs) and memory B cells (Bmems). (TIF)

S4 Fig Effect of Vac-1 on CDI in mouse model. (TIF)

S1 Table Strains and Plasmids used in this study. (PDF)
