# Supplementary material for: The impact of TcdB conformational dynamics on vaccination against Clostridioides difficile
Source: PLoS Pathog. 2026 Sep 18;22(9):e1014627. doi: 10.1371/journal.ppat.1014627 (PMC13600615; doi:10.1371/journal.ppat.1014627)
Supplement: Supplementary file 1 [file ppat.1014627.s005.pdf]

Table S1. Strains and plasmids used in this study.

Plasmid or strain	Description	Source or reference
***C. difficile* strains**		
R20291	Clinical isolate of *C. difficile*	gift from Dr. Casey Theriot, North Carolina State University
***B. megaterium* strains**		
WH320	Protein production strain	MoBiTec, Göttingen, Germany
**Plasmids**		
pC-His1622-tcdB1	Expression or recombinant TcdB1	gift from Dr. Borden Lacy, Vanderbilt University Medical Center
pC-His1622-tcdB2	Expression of recombinant TcdB2	(1)
pTMS017	Expression of recombinant $TcdB2_{\Delta 2291\text{-}2366}$	(2)
pTMS023	Expression of recombinant Vac-1	This Study
pTMS024	Expression of recombinant $TcdB2_{D270N}$	This Study; originally (3)
pTMS025	Expression of recombinant $TcdB2_{C698S}$	This Study; originally (3)
pC-His1622-$tcdB2_{\Delta 1769\text{-}1787}$	Expression of recombinant $TcdB2_{\Delta 1769\text{-}1787}$	(4)

1. Larabee, J. L. et al. Exposure of neutralizing epitopes in the carboxyl-terminal domain of TcdB is altered by a proximal hypervariable region. *J Biol Chem* **290**, 6975–6985 (2015).
2. Kempher, M. L. et al. A sequence invariable region in TcdB2 is required for toxin escape from *Clostridioides difficile*. *J Bacteriol* **206**, e0009624 (2024).
3. Chumbler, N. M. et al. *Clostridium difficile* Toxin B causes epithelial cell necrosis through an autoprocessing-independent mechanism. *PLoS Pathog* **8**, e1003072 (2012).
4. Bland, S. J. et al. Deletion of a 19-amino-acid region in *Clostridioides difficile* TcdB2 results in spontaneous autoprocessing and reduced cell binding and provides a nontoxic immunogen for vaccination. *Infect Immun* **87** (2019).
